# A robust potency assay highlights significant donor variation of human mesenchymal stem/progenitor cell immune modulatory capacity and extended radio-resistance

**DOI:** 10.1186/s13287-015-0233-8

**Published:** 2015-12-01

**Authors:** Nina Ketterl, Gabriele Brachtl, Cornelia Schuh, Karen Bieback, Katharina Schallmoser, Andreas Reinisch, Dirk Strunk

**Affiliations:** Experimental and Clinical Cell Therapy Institute, Spinal Cord Injury and Tissue Regeneration Center, Paracelsus Medical University, Salzburg, Austria; Institute of Transfusion Medicine and Immunology, Medical Faculty Mannheim, Heidelberg University, Red Cross Blood Service Baden-Württemberg-Hessen, Mannheim, Germany; Department of Transfusion Medicine and Spinal Cord Injury and Tissue Regeneration Center, Paracelsus Medical University, Salzburg, Austria; Institute for Stem Cell Biology and Regenerative Medicine, Stanford School of Medicine, Stanford University, Stanford, CA USA

**Keywords:** Potency assay, Immune response modulation, Mesenchymal stem/progenitor cells, T cells

## Abstract

The inherent immunomodulatory capacity of mesenchymal stem/progenitor cells (MSPCs) encouraged initiation of multiple clinical trials. Release criteria for therapeutic MSPCs cover identity, purity and safety but appropriate potency assessment is often missing. Reports on functional heterogeneity of MSPCs created additional uncertainty regarding donor and organ/source selection. We established a robust immunomodulation potency assay based on pooling responder leukocytes to minimize individual immune response variability. Comparing various MSPCs revealed significant potency inconsistency and generally diminished allo-immunosuppression compared to dose-dependent inhibition of mitogenesis. Gamma-irradiation to block unintended MSPC proliferation did not prohibit chondrogenesis and osteogenesis in vivo, indicating the need for alternative safety strategies.

**Electronic supplementary material:**

The online version of this article (doi:10.1186/s13287-015-0233-8) contains supplementary material, which is available to authorized users.

## Introduction

High expectations in the therapeutic potential of mesenchymal stem/progenitor cells (MSPCs) led to the initiation of >500 clinical trials mainly testing the major histocompatibility (MHC) antigen-independent immunomodulatory and trophic capacity of autologous and allogeneic MSPCs propagated from various organs (558 studies registered by 4 November 2015; www.clinicaltrials.gov search term = mesenchymal stem cell) [1, 2]. The general concept of regenerative stem cell therapy is based on evidence that stem/progenitor cells can contribute to the regeneration of damaged organs, improve their function or even cure diseases [3, 4]. Therapeutic application of culture-expanded MSPCs was initiated >20 years ago to support hematopoietic stem cell engraftment [5]. Repair of large bone defects by bone marrow (BM)-MSPCs [6] and immune suppressive therapy for steroid-refractory graft-versus-host disease [7, 8] encouraged an extended spectrum of regenerative and immunomodulatory applications. Measurable effects despite lack of sustained engraftment of transplanted MSPCs led to the current view favoring trophic and immunomodulatory MSPC functions to be decisive for therapeutic effects [1, 2, 5].

However, still incomplete mechanistic insight and absence of predictive biomarkers or potency assays constrain projectable clinical applicability of advanced MSPC therapies [9]. Selection of suitable MSPC donors, organ sources, propagation procedures, and cell doses, as well as mode and timing of application is complicated by functional MSPC heterogeneity within a given tissue and also between different organs [10, 11]. Contradictory data demonstrating superiority of certain MSPCs and impaired efficiency of freshly thawed compared to culture-derived cells may at least in part depend on variable readout assay formats [12–16]. This added a level of uncertainty to the ongoing clinical trials that frequently employ off-the-shelf MSPCs which are thawed upon application without appropriate potency information at the bedside. Robust ‘potency assays as companion to trials’ are thus urgently needed [9]. Inspired by previous attempts of pooling therapeutic MSPCs [17], the pooling of cord blood leukocytes for transplantation [18] and our longstanding experience with pooled human platelet lysate (pHPL) as prevailing animal serum replacement for efficient cell propagation [19–22], we hypothesized that pooling both responder T cells as well as inhibitory MSPCs may help to overcome the variability of individual donor-derived cells in a potency assay.

Here we introduce a robust potency assay using inhibition of pooled polyclonal T-lymphocyte proliferation (i) after mitogen or antibody stimulation as well as (ii) due to allo-antigen-driven mixed leukocyte reaction (MLR). Pooled organ-specific reference MSPCs were used as a nominator to qualify individual donor MSPC potency for inhibiting mitogen-induced as well as MLR-driven T-cell proliferation in parallel for direct comparison. Leukocytes from ten donors were pooled to counterbalance individual immune response variability. Cells were pre-labeled with carboxyfluorescein and cryopreserved in aliquots for instant and reproducible off-the-shelf use. Based on clinical practice, MSPCs were tested immediately after thawing (off-the-shelf) or after an approximate 72-hour ‘rescue culture’ for their potency to inhibit both polyclonal and allogeneic T-cell proliferation. MSPCs from five random human BM, white adipose tissue (WAT) and umbilical cord (UC) donors were analyzed individually or as a pool. A supplementary safety measure was intended by testing 30 Gy irradiation of the MSPCs to minimize the risk of continued proliferation after application.

Here we show that all MSPCs displayed significant dose-dependent suppression of T-cell mitogenesis. Despite significant individual variation we observed comparable overall suppression of mitogen-induced T-cell proliferation by the different organ-derived MSPCs. The inhibition of a multidirectional allo-response within the pool of ten peripheral blood mononuclear cell (PBMC) preparations was less efficient by many, but not all, of the MSPCs, presumably indicating a particular level of potency variability. Pooling multiple donor-derived MSPCs compensated inter-individual variation and allowed us to compare individual donor MSPC immunosuppression potency with their organotypic reference MSPC pool. Irradiation did not significantly hamper MSPC functionality, but a note of caution has to be drawn due to the maintained chondrogenic and osteogenic differentiation of 30 Gy irradiated BM-MSPCs in vivo.

## Methods

### Cell isolation, culture, and immunophenotyping

Approval was obtained for human cell and tissue sample collection from the Institutional Review Board (protocols 19–252, 18–243, 21–060, 19–284 and 415-E/1776/4-2014, Ethics Committee of the province of Salzburg). Adult samples were collected in accordance with the Declaration of Helsinki after written informed consent from healthy volunteers. Donor age is documented in Additional file 1 (Table S1). UC samples were collected after written informed consent by the mother-to-be obtained prior to delivery of full-term pregnancies. MSPCs from BM and UC were isolated and expanded under animal serum-free conditions using pHPL to replace fetal bovine serum and their purity, identity and viability was characterized by flow cytometry as previously described [21, 23, 24]. WAT-MSPCs were obtained from stromal vascular fractions of WAT and characterized as published [25]. MSPCs were tested either directly after thawing and 2 × washing (off-the-shelf) or after an approximate 72-hour rescue culture to revert a putative freeze/thaw-induced functional damage [14]. PBMCs were isolated by density centrifugation from random donor buffy coats as described [20].

### T-cell proliferation assay

Immunomodulatory potency of MSPCs was determined as described [20] with modifications as follows. PBMCs from ten random donors (i.e., 1 × 10^10^ PBMCs derived from ten buffy coats) were pooled in 500 mL pre-warmed phosphate-buffered saline (37 °C; Sigma) for staining with carboxyfluorescein succinimidyl ester (CFSE, 2 μM, 15 minutes, 37 °C; Sigma) in the dark at a cell density of 2 × 10^7^ PBMCs/mL. The reaction was stopped by adding an equal amount of RPMI-1640 medium (Sigma) supplemented with 10 % human blood group AB serum. After washing twice in RPMI/10 % AB serum, 200 aliquots of 5 × 10^7^ CFSE-labeled PBMCs could be cryopreserved in liquid nitrogen as described [26] for off-the-shelf use as reference responders in multiple subsequent experiments. Based on preliminary titration experiments comparing various PBMC and MSPC numbers in different cell culture plate formats, 3 × 10^5^ CFSE pre-labeled PBMCs resuspended in RPMI-1640 supplemented with 10 % pHPL, 2 IU/mL preservative-free heparin (Biochrom), 2 mM L-glutamine, 10 mM HEPES (Gibco), 100 IU/mL penicillin and 100 μg/mL streptomycin (Sigma) were plated per well in triplicate in 96-well flat-bottomed plates (Corning). T-cell proliferation was determined in the absence or presence of 5 μg/ml phytohemagglutinin (PHA; Sigma) or CD3/CD28 beads (1:1 ratio; Invitrogen) following manufacturer’s instructions with or without graded numbers of MSPCs (250 μL total volume per well) in limited threefold dilution as indicated in the results section. MSPCs were irradiated (30 Gy; Cs-139 Source, Blobeam 2000 Gamma irradiator, GSM) as indicated in the results section. Corresponding paired MSPC samples were mock-irradiated (i.e., were transported together with the irradiated cells but left standing outside the irradiation device at room temperature).

For transwell experiments, randomly selected samples from one of each BM-, WAT- and UC-MSPC donors were seeded in 24-well plates (Corning) in RPMI-1640 assay medium at different ratios as indicated in the results section (3 × 10^5^, 1 × 10^5^, 3 × 10^4^, 1 × 10^4^ cells per well, total volume 500 μl, to reach a MSPC:PBMC ratio of 1:1, 1:3, 1:10 and 1:30, respectively) at 37 °C. RPMI assay medium (100 μL) either with PHA (final concentration 5 μg/ml for day 4 mitogenesis measurements) or without additional stimulus (for untreated samples and day 7 MLR measurements) was added per 24-well to get a total volume of 600 μl after 24 hours. Transwell polyester membrane cell culture inserts (0.4 μm pore size, 0.33 cm^2^ matching the growth area of a 96-well plate well; Corning) were transferred to all 24-wells before adding 100 μl CFSE-labeled pooled PBMCs (3 × 10^5^ cells/transwell insert) either with or without PHA stimulus (final concentration 5 μg/ml). In parallel, the standard immune modulation assay allowing cell–cell contact as described above was performed with aliquots of the same cell populations in flat-bottomed 96-well plates. Both assay formats were cultured for 4 and 7 days before the proliferation of viable CD3^+^ cells was analyzed via flow cytometry. All cultures were performed in humidified ambient air incubators (Binder CB210) at 37 °C and 5 % CO_2_.

T-cell proliferation was determined using a Gallios 10-color flow cytometer and the Kaluza G1.0 software (both Coulter). Viable 7-aminoactinomycin-D-excluding (7-AAD^−^; BD Pharmingen) CD3-APC^+^ (eBioscience) T cells were analyzed after 4 to 7 days. Proliferation kinetics and population distribution were analyzed using Modfit 4.1 software (Verity). A MLR is known to occur as a consequence of pooling multiple individual donor-derived PBMC preparations. Taking advantage of this phenomenon, allogeneic MLR-driven polyclonal T-cell proliferation was determined in addition in the absence or presence of serially threefold diluted numbers of MSPCs.

### Bone formation in vivo

All mouse experiments were approved by the Institutional Animal Care and Use Committee (Stanford Administrative Panel on Laboratory Animal Care no. 22264) and in adherence to the US National Institutes of Health’s Guide for the Care and Use of Laboratory Animals. Previous results demonstrated that BM-MSPCs can form a human bone and hematopoietic marrow niche via a vascularized cartilage intermediate when injected subcutaneously into immunodeficient NOD.Cg-Prkdc^scid^ Il2rg^tm1Wjl^/SzJ (NSG) mice (6–18 weeks old; Jackson Laboratory) [27]. Using this model, we tested whether 30 Gy irradiated BM-MSPCs can differentiate (as can native non-irradiated BM-MSPCs) in vivo essentially as described [27]. Prior to application, BM-MSPCs were either irradiated (30 Gy, Caesium-137 irradiator) or left non-irradiated. Cells were resuspended in matrigel-equivalent matrix (Millipore) and injected subcutaneously (four injections per animal, 2 × 10^6^ cells per injection, two injections with irradiated and non-irradiated cells in the left and right flank of each animal, respectively). Differentiation of cells within the plugs was analyzed after 6, 9 and 12 weeks as described [27].

### Statistical analysis

Values are presented as mean ± standard deviation (SD). Prism version 6.00 for Windows (GraphPad Software) was used for one-way analysis of variance statistical analysis. *p* values < 0.05 were considered significant.

## Results and discussion

**Fig. 1:**
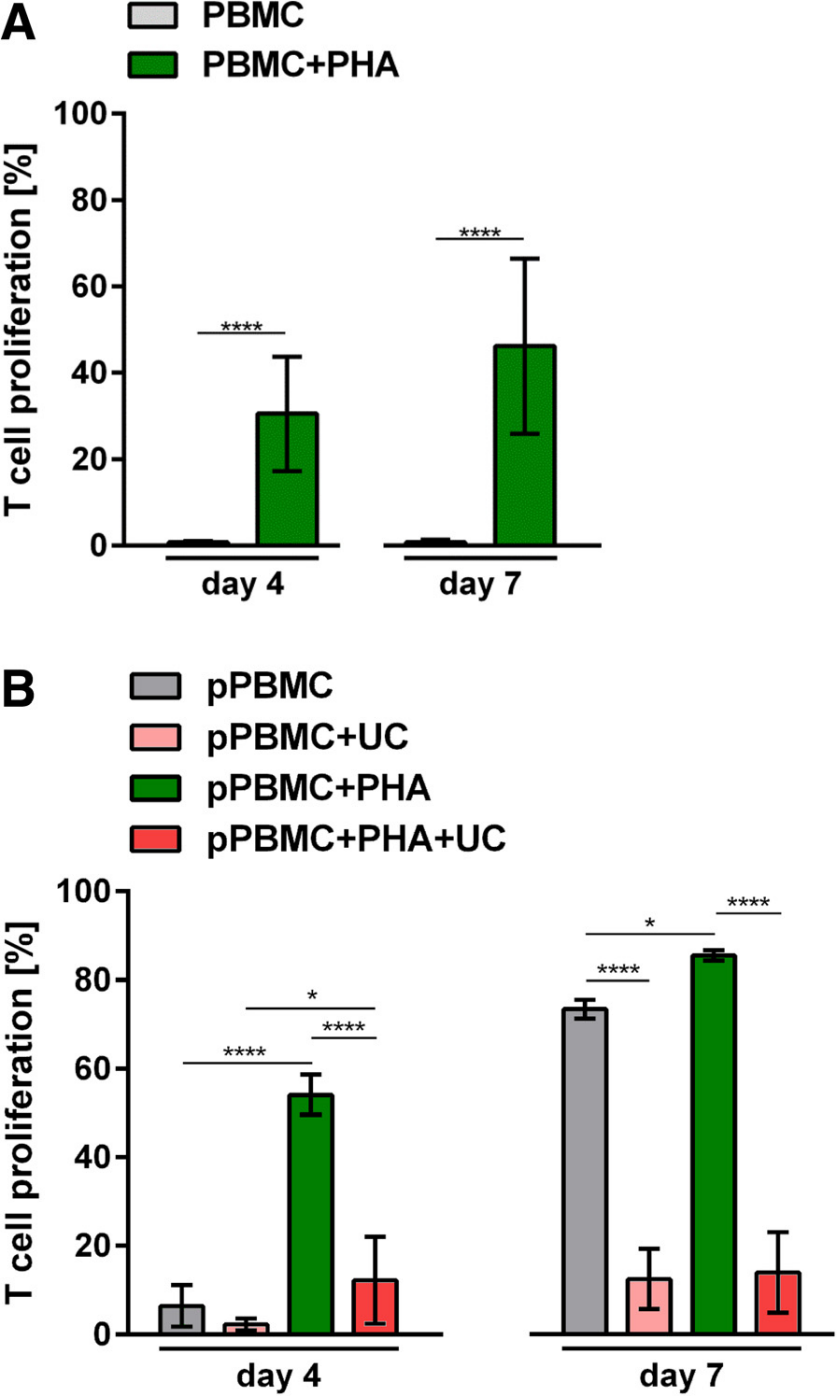
Individual or pooled donor polyclonal T-cell proliferation. **a** Mean ± SD proliferation of five random single donor buffy coat-derived CFSE-labeled peripheral blood mononuclear cells (*PBMCs*) in the absence (*grey bars*) or presence of phytohemagglutinin (+PHA, *green bars*) after 4 or 7 days indicating significant donor variation. **b** Simultaneous inhibition of mitogenic and alloimmunogenic T-cell proliferation by umbilical cord (*UC*)-derived MSPCs was tested. CFSE pre-labeled cryopreserved pooled PBMCs (*pPBMCs*) were seeded in the absence of MSPCs without (*grey bars*) or with PHA (*green bars*) or in the presence of UC-MSPC without (*light red bars*; inhibition of allo-MLR) or with PHA (*red bars*; inhibition of additional mitogenesis) at an MSPC:PBMC ratio of 1:3. The time course of proliferation between days 4 to 7 is shown in Additional file 2 (Figure S1C). Mean ± SD results from representative experiments (n = 5 in **a** and **b**). Significant differences are indicated (*p < 0.05; **p < 0.01; ***p < 0.001; ****p < 0.0001)

**Fig. 2:**
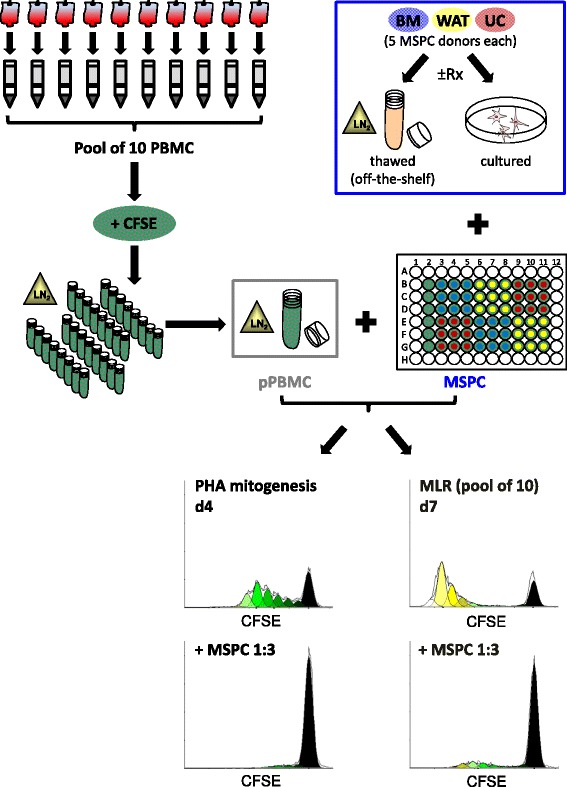
Illustrated potency assay strategy. Ten randomly obtained buffy coats from healthy donors can be processed in parallel to isolate approximately 1 × 10^9^ peripheral blood mononuclear cells (*PBMCs*) per donor, pooled, labeled with carboxyfluorescein (*CFSE*) and cryopreserved in appropriate aliquots (e.g., 200 aliquots of 1 × 10^7^ pre-labeled pooled PBMCs (*pPBMC*)) for subsequent off-the-shelf use as responder cells in the potency assay. Individual mesenchymal stem/progenitor cells (*MSPC*) from donor or organ origin of choice (e.g., bone marrow (*BM*), white adipose tissue (*WAT*), umbilical cord (*UC*); color code corresponding to Fig. 1) can be tested off-the-shelf or after rescue culture (with or without gamma irradiation (*±Rx*)) to test their potency to inhibit mitogen (e.g., PHA)-driven pPBMC proliferation until day 4 (*d4*), or to inhibit the allogeneic mixed lymphocyte reaction (*MLR*) of the same pPBMC batch until day 7 (*d7*). Representative CD3^+^ T-cell proliferation kinetics (Modfit analysis) in the absence (*top histograms*), or presence of regulatory MSPCs (*bottom histograms*) indicating maximum number of proliferated populations (*top histograms*) and the effect of MSPC-mediated inhibition of T-cell proliferation (*bottom histograms*) are shown

In an initial series of experiments we confirmed that individual buffy coat-derived PBMCs displayed a significant variability of T-cell proliferation in response to polyclonal PHA stimulation (Fig. 1 and Additional file 2: Figure S1A). This is also in accordance with recently published data showing >50 % variation of individual donor T-cell proliferation after polyclonal stimulation [28]. This confirmed that individual responder cells do not allow for reproducible monitoring of MSPC immunosuppression potency. Pooling ten random donor-derived PBMCs resulted in a significant time-dependent MLR beyond day 4 and increasing until day 7 due to cross-stimulation of the mixed PBMCs in the absence of additional external stimuli. Mitogen (PHA) or CD3/CD28 crosslinking-driven polyclonal responses at day 4 were still significantly higher than the MLR (Additional file 2: Figure S1B). We selected PHA-driven polyclonal mitogenesis at day 4 as well as allogeneic MLR-based polyclonal T-cell proliferation at day 7 as a dual strategy to test the potential of different MSPCs for inhibition of T-cell proliferation. Validating this assay format we proved that UC-MSPCs from a randomly selected donor could sufficiently inhibit both the mitogenesis and the allogeneic MLR of pooled PBMCs in a time course testing 4 to 7 days of assay duration (Additional file 2: Figure 1B and S1C). The gating strategy based on these experiments is shown in Additional file 3 (Figure S2). A schematic illustrated summary of the robust dual potency assay format is shown in Fig. 2. Using this assay format the PHA-driven proliferation may well be replaced by using other stimuli of B cells and natural killer cell proliferation combined with addition of CD19 and CD56 antibodies.

MSPCs from various tissues can display substantial differences in their capacity to inhibit immune responses despite common immunophenotype and tri-lineage differentiation capacity [13, 29]. MSPCs used in this study were previously isolated from BM, WAT and UC and did not reveal significant phenotypic differences as published [27]. Their tri-lineage osteo-, chondro- and adipogenic differentiation was also confirmed previously indicating quantitative differences resulting from an organ-specific epigenomic signature [27]. When testing fifteen individual BM-, WAT-, and UC-MSPC donors (five donors per source) we observed a significant inhibition of pooled polyclonal T-cell mitogenesis by MSPCs from all three sources at 1:3 and 1:10 ratio. At a 1:30 ratio all MSPCs except cultured BM-MSPCs significantly inhibited T-cell mitogenesis (Additional file 4: Figure S3A–C). These data confirmed previously reported evidence for inferior immunosuppressive potency of BM-MSPCs [12, 13]. Whether this lack of immunosuppression of the BM-MSPCs tested in different centers may also translate to reduced efficiency of BM-MSPCs compared to MSPCs from other sources in vivo will be indicated soon as a result of several ongoing clinical trials.

**Fig. 3:**
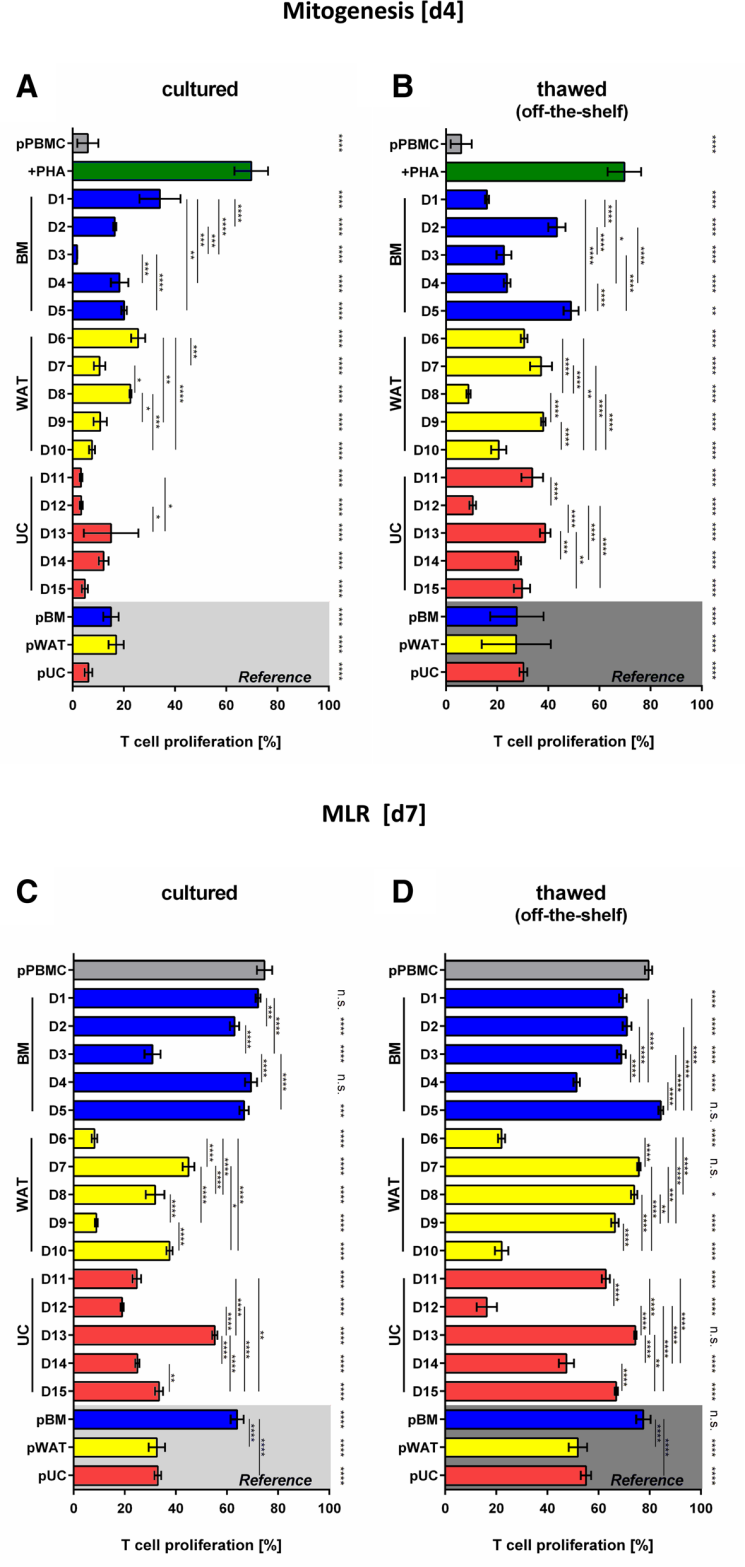
Pooled pre-labeled PBMC and pooled reference MSPC make a robust assay format to readout inhibition of T-cell proliferation in an off-the-shelf potency assay. **a**, **b** Pooled carboxyfluorescein pre-labeled random donor peripheral blood mononuclear cell (*pPBMC*) aliquots seeded in triplicate show a highly significant mitogen-induced proliferation (phytohemagglutinin (*PHA*); *green bars*) compared to minimum proliferation of the unstimulated pPBMC seeded off-the-shelf in the absence of PHA (*dark grey bar*) at day 4 (*d4*). Triplicates of pMSPC composed of cells from five each random donors (*D1*–*D15*) of bone marrow (*BM*; *blue bars*), white adipose tissue (*WAT*; *yellow bars*) and umbilical cord (*UC*; *red bars*) were used as an organotypic MSPC reference (*grey areas*) to determine organ-specific highly significant inhibition of mitogen-induced T-cell proliferation compared to individual **a** cultured or **b** freshly thawed individual MSPC from five donors per organ. **c, d** At day 7 (*d7*), the potent allo-response of pPBMC (*grey bars*; for time course titration see Fig. 1b, c) in the absence of external stimulation was significantly inhibited by some but not all individual MSPCs compared to the organotypic reference pMSPC (*grey areas*; same donors and identical color code as in **a**, **b**). Differences between **c** cultured and **d** freshly thawed individual or pooled MSPCs were more prominent compared to the inhibition of mitogenesis (in **a**, **b**). Significant inhibition of day 4 mitogen-induced (**a**, **b**) and day 7 mixed leukocyte reaction (*MLR*)-induced (**c**, **d**) pooled T-cell proliferation is indicated at the right margin of the graphs; significance of individual donor comparison is indicated by vertical lines. Mean ± SD results at an MSPC:PBMC ratio of 1:3 are shown. *p < 0.05; **p < 0.01 ***p < 0.001; ****p < 0.0001

Comparing the individual MSPC preparations which were all propagated under identical conditions in alpha-MEM/10 % pHPL and cryopreserved at early passage for subsequent use, a profound variation in their potency to inhibit T-cell proliferation became obvious. It may be hypothesized that for selected donors a freeze/thaw-related damage resulted in a profound loss of potency (Fig. 3). We speculate that, in addition to the obvious donor variation, differences during cell processing and in the freezing/thawing protocols may contribute to this phenomenon [13–16]. This may also result from heterogeneity of MSPCs derived from the same organ-of-origin of different donors in different laboratories [16]. In any case the established variability of different MSPC preparations is emphasizing the need for a robust potency assay.

Next we aimed to determine whether freshly thawed compared to cultured individual donor-derived MSPC inhibitory effectiveness can be qualified relative to a pool of reference cells. Therefore, we tested their potency to inhibit polyclonal T-cell proliferation either individually or combined as organotypic reference pools. Results revealed significant differences between individual donors compared to their organotypic reference (Fig. 3a, b). Particular MSPCs from BM (donor one, D1) and WAT (donor eight, D8) displayed higher inhibitory potential when tested immediately after thawing (off-the-shelf) compared to corresponding cells tested after a rescue culture period. MSPCs from other donors showed impaired inhibitory potency thus reproducing published results [14]. Most but not all aliquots of the same MSPCs also significantly inhibited allogeneic MLR-induced proliferation of the same PBMC pool until day 7 in the absence of mitogen. Numerous MSPCs were less efficient in inhibiting the MLR than the PHA-driven mitogenesis. Some (WAT donors six and nine and UC donor 12; D6, D9 and D12, respectively) showed at least comparable inhibitory potency. Although significant individual variability was observed, both conditions indicated superiority of UC- and WAT-MSPCs over BM-MSPCs (Fig. 3c, d). Such data may be interpreted in favor of including potency assays in the release criteria of advanced cell therapy medicinal products to better select MSPC donors (in the case of third party or allogeneic MSPC products) and processing methodology (i.e., off-the-shelf use versus rescue culture of cryopreserved MSPCs).

**Fig. 4:**
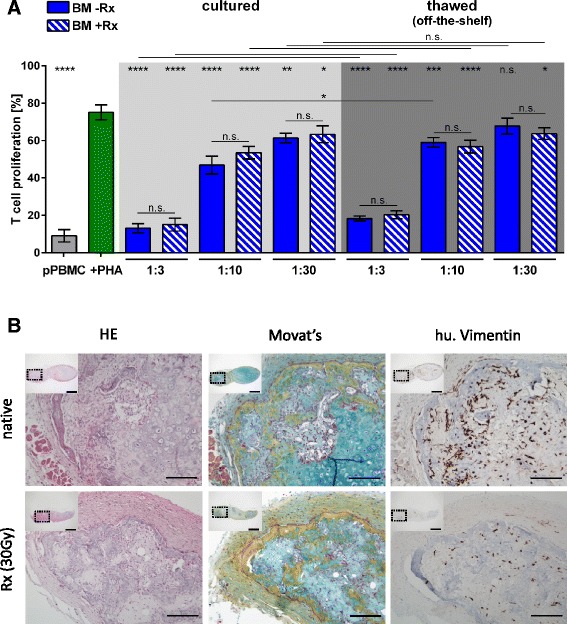
Irradiated MSPC maintain their immunomodulatory potency in vitro and their differentiation capacity in vivo. **a** Direct comparison of the inhibition of phytohemagglutinin (*PHA*)-induced T-cell proliferation (*green bar*) by non-irradiated bone marrow (BM)-MSPCs (*-Rx*; *blue bars*) versus 30 Gy irradiated BM-MSPCs (*+Rx*; *hatched blue bars*) immediately after thawing (off-the-shelf use; *dark grey area*) or after a 3-day rescue culture (*light grey area*) showed no significant difference at the three ratios as indicated (*p < 0.05; **p < 0.01; ***p < 0.001; ****p < 0.0001). *Grey bar* shows mean ± SD of unstimulated pooled T-cell proliferation. One representative experiment out of two is shown. **b** Representative histologic analysis of ectopic ossicles derived from native (non-irradiated, *upper pictures*) and irradiated (*Rx*; 30 Gy, *lower pictures*) BM-MSPC (n = 6 per group) 6 weeks after subcutaneous transplantation into immunocompromized NSG recipient mice. Bone formation via a vascularized cartilage intermediate was evident in hematoylin and eosin (*HE*; *left panels*) as well as Movat’s pentachrome (*Movat*; *middle panels*) staining. Vimentin staining (*right panels*) indicating persistence of reticular stromal cells (MSPCs) within the ectopic ossicles which showed infiltration by (human (*hu.*) Vimentin-negative) murine hematopoiesis as described previously only for native (non-irradiated) BM-MSPCs [27]. Scale bars are 100 μm in main histophotographs and 1 mm in inserts (showing overview of a section through the entire ossicle; *dotted rectangles* indicate the regions from where the magnified main pictures were derived). *n.s.* Not significant, *pPBMC* Pooled peripheral mononuclear cell

To address the question whether the T-cell inhibitory function of MSPCs in this assay format is cell contact-dependent we performed additional experiments directly comparing randomly selected MSPCs from BM, WAT and UC in parallel either in direct cell–cell contact with the pooled PBMCs or in transwell cultures separating MSPCs (in the lower compartment) from PBMCs (in the transwell insert). Results revealed significant inhibition of PHA-induced T-cell mitogenesis independent of cell–cell contact. Inhibition of T-cell mitogenesis was significantly more efficient at PBMC:MSPC ratios of 1:1 and 1:10 at direct cell–cell contact. The allogeneic MLR resulting from cross-stimulation of the pooled PBMCs in the same assay format at day 7 was more significantly inhibited by UC-MSPCs at most PBMC:MSPC ratios. Inhibition of the MLR by BM- and UC-MSPCs was less efficient thus in part resembling data in Fig. 3 and published results. Also in the allogeneic MLR situation, depending on the PBMC:MSPC ratio, a cell–cell contact-independent inhibition of T-cell proliferation was observed (Additional file 5: Figure S4). Whether the simple standard assay testing MSPCs in direct contact with PBMCs, rather than the more complex transwell assay, might be able to predict the therapeutic MSPC potency in vivo needs to be determined in prospective clinical trials. Furthermore, we evaluated if 30 Gy gamma-irradiation of freshly thawed or cultured BM-MSPCs can be introduced as a putative safety measure before cells will be applied in vivo. Our results showed that irradiation did not influence their immunosuppressive potency (Fig. 4a). Interestingly, 30 Gy irradiation did not affect the differentiation potential of BM-MSPCs. We analyzed cartilage and bone formation of irradiated and non-irradiated BM-MSPCs in NSG mice and found that despite irradiation human Vimentin^+^ BM-MSPCs survived for up to 12 weeks in the immunocompromized animals and maintained their potential to form bone via a vascularized cartilage intermediate as recently described [27]. Hematoxylin and eosin as well as Movat’s pentachrome staining clearly demonstrated hypertrophic cartilage, osteoid and mineralized bone formation accompanied by immigration of murine marrow (Fig. 4b). This outcome extends a recent observation by Bianco and coworkers [30] showing that cartilage constructs generated from human BM-MSPCs ex vivo maintain their bone formation potential even when irradiated before transplantation into immunodeficient mice. These authors elegantly demonstrated that cartilage differentiation of BM-MSPCs in vitro is reversible and can be reverted, despite irradiation, in vivo, resulting in the generation of stromal hematopoietic niche-forming cells [30]. The goal of our experiments, in this study, was to determine whether BM-MSPCs are still capable of initiating patent chondrogenesis and subsequent osteogenesis after irradiation in advance of differentiation. Our observation that 30 Gy irradiation of human BM-MSPCs ex vivo did not impair cartilage and bone formation in vivo does not exclude the possibility that Vimentin^+^ stromal niche elements observed particularly in areas of hematopoiesis immigration could be derived from intermediate chondrocytes. A note of caution thus needs to be drawn regarding applicability of irradiated BM-MSPCs which might maintain their differentiation capacity if applied solely for immunomodulatory purposes. Whether unintended differentiation (as shown in this study and others [30, 31]) has to be considered a firm risk after systemic application needs to be assessed separately. Another relevant consequence of ex vivo irradiation may be premature MSPC senescence resulting in impaired immunomodulatory efficiency in vivo [32].

The pooling of five MSPC and ten PBMC donor samples to compose the reference pools and the universal responder pooled PBMCs, respectively, to simultaneously measure mitogenesis and MLR was based on practical considerations. It may be speculated that increasing the number of different MSPCs per reference MSPC pool may even improve assay performance. Pre-selection of highly potent MSPCs as a reference could result in excluding a profound number of donors due to apparently inferior potency. From a practical point of view, using randomly selected MSPC donors for composing a reference MSPC pool may display a realistic reference. The use of a pool of ten PBMC donors proved to be practicable based on pilot experiments to achieve a high number of test aliquots and still maintained the discrimination of mitogenesis and MLR at days 4 and 7, respectively (Additional file 2: Figure S1B). Processing ten buffy coats to recover approximately 1 × 10^10^ PBMCs which could be efficiently labeled with CFSE in a volume of 500 mL and produced 200 aliquots of 5 × 10^7^ pooled pre-labeled test PBMCs was shown to be practicable (Fig. 2). In a total of 35 experiments the pool of ten PBMCs showed low variability (mean ± SD, 66.05 ± 11.38 % PHA-induced day 4 and 73.04 ± 5.44 % MLR-induced day 7 T-cell proliferation, respectively). Reducing the number of PBMC donors within a pool will reduce the power of the multivalent MLR and thus help to adjust the strength of the allo-response to be inhibited by MSPCs or other regulatory cells. Our observation that some MSPC strains display an equal potency of inhibiting a strong multivalent MLR may be useful to select potent MSPC donors for the treatment of strong allo-responses, e.g., during severe graft-versus-host reactions. Current data do not allow speculating whether a defined number of MHC mismatches between PBMC donors or a particular strength of an MLR is required to predict MSPC potency in a therapeutic setting.

MSPCs from different sources have been used for treating thousands of patients suffering from a plethora of diseases. Lack of robust potency assays and a still-limited mechanistic insight into their mode of action evidently hamper the development of optimized and efficient MSPC therapy strategies. Our prime future goal will be prospectively testing the validity of this assay in ongoing clinical trials for the treatment of severe therapy-refractory graft-versus-host disease and multiple sclerosis. Determining the predictive value of an immunosuppression potency assay will require correlation of assay results with treatment outcome. Monitoring immunosuppressive effects of MSPCs may not be restricted to immunosuppressive therapies. Given the inherent immunomodulatory capacity of MSPCs, such a potency assay may also help to predict immunosuppressive side effects of MSPCs when used for co-transplantation to enhance hematopoietic cell engraftment or during other types of non-immunologic regenerative therapies. Inhibition of thymic reconstitution and reduced immunoglobulin levels has recently been found to be associated with MSPC co-infusion at the time of umbilical cord blood hematopoietic cell transplantation [33]. It is still not known whether variability of the MSPC immunosuppressive potential correlates with inconsistencies of their three-lineage differentiation [27]. It is also not clear whether the level of cytokine and trophic factor secretion by MSPCs correlates with treatment outcome [13]. These points need to be addressed in future analyses with a particular focus on profiling soluble factors known to be involved in MSC-derived immune response modulation including interferons, chemokines and pro-apoptotic molecules, among others. Based on our previous observation indicating that epigenetics can distinguish MSCs from different sources it will also be interesting to precisely study promotor methylation status of key immunomodulatory molecules to address questions of epigenetic regulation of immunomodulation [27].

A thorough understanding of the multiplicity of stromal niche cell functions will also be required for better predicting clinical effects as well as side effects of these otherwise easily expandable and widely available cells. Once established, appropriate potency assays may also help to gain more detailed insight into the mechanisms underlying the largely unpredictable variability of MSPC function, eventually resulting in more efficient advanced cell therapy products. The potency assay introduced herein has intentionally been set up to function entirely animal serum-free using pHPL for replacing fetal bovine serum. We have previously described an unaffected viral T-cell immunity when using BM-MSPCs to inhibit T-cell proliferation in vitro [34]. Provided that this also occurs in vivo the propagation of MSPCs in pHPL rather than in bovine serum may be preferred for clinical use.

We propose using such a standardized potency assay as a reference to validate MSPC effectiveness. Selection of pre-tested highly potent individual or pooled MSPCs may also offer a valid alternative to individualized cell therapy strategies. Gamma irradiation, while considered an established safety measure inhibiting unintended proliferation, was not sufficient to prohibit chondrogenic and osteogenic MSPC differentiation thus indicating the need to identify alternative strategies for blocking unintended differentiation.

## Conclusions

- A robust immunosuppression potency assay has been established using CFSE pre-labeled pooled and cryopreserved PBMCs which can be tested off-the-shelf for mitogenesis-driven lymphocyte proliferation and MLR. The inhibitory potential of individual MSPCs was compared to pooled MSPCs as a reference normalizing donor variation in this combined assay format.
- Fifteen individual test MSPCs from three organs displayed significant donor-dependent variability in their immunosuppressive potency. UC- and WAT-MSPCs were more potent than BM-MSPCs at inhibiting multidirectional allo-MLR of pooled PBMCs and also at inhibiting pooled T-cell mitogenesis at a higher PBMC:MSPC ratio (1:30). A proportion of MSPCs was sensitive to freeze/thaw damage extending published results and indicating validity of this assay.
- Gamma irradiation did not hamper MSPC immunosuppression capacity. A note of caution was raised by the observation that irradiated BM-MSPCs maintained their capacity to differentiate along chondrogenic and osteogenic lineages in vivo.

## Additional files

Additional file 1: Table S1. MSPC Donor age and origin. (DOC 63 kb) Additional file 2: Figure S1. Time course of polyclonal T cell proliferation. (A) Random donor single buffy coat-derived carboxyfluorescein (CFSE)-labeled peripheral blood mononuclear cells (PBMC 1–5) were plated in triplicate and cultured in the absence (PBMC, grey bars) or presence of phytohemagglutinin (+PHA, green bars) for 4 or 7 days resulting in significantly donor-variable maximum proliferation. (B) Pooled PBMC from ten random BC donors (pPBMC) were plated in triplicate and cultured for 3 to 7 days either without additional stimulation (grey bars) or in the presence of PHA (green bars) or CD3/CD28-coated beads (open bars) representing standard stimuli of T-cell proliferation. The mixed leucocyte reaction in the ten-donor PBMC pool was negligible until days 3 and 4 but constantly increasing thereafter representing a prototypic allo-response. (C) To test the hypothesis that pooling of CFSE pre-labeled cryopreserved responder PBMCs allows for the simultaneous readout of inhibition of the mitogenic and alloimmunogenic T-cell proliferation by mesenchymal stem/progenitor cells (MSPC), pooled CFSE pre-labeled PBMCs were seeded in the absence of MSPCs without (grey bars) or with PHA (green bars) or in the presence of third party umbilical cord-derived (UC)-MSPC without (light red bars; inhibition of allo-response) or with PHA (red bars; inhibition of additional mitogenesis) at an MSPC:PBMC ratio of 1:3. Time course of proliferation and inhibition of proliferation, respectively, was measured by flow cytometry depicting the percentage of proliferating CD3^+^ T cells. Mean ± SD results from representative experiments (n = 5 in A and C; n = 3 in B). Significant difference are indicated (**p* < 0.05; ***p* < 0.01; ****p* < 0.001; *****p* < 0.0001). (DOC 148 kb) Additional file 3: Figure S2. Gating strategy for proliferation analysis. (A) For accurate analysis of proliferating T cells we combined (i) doublet discrimination by forward scatter width (FS-W) vs. sides scatter area (SS-A) plotting with (ii) DNA dye exclusion capability of viable 7AAD-excluding (negative) cells that display mononuclear FS-A characteristics and (iii) CD3 –APC reactivity to identify T cells. One representative (out of >30 performed assays) is depicted. (B) Representative (iv) control cells (pooled peripheral blood mononuclear cells, pPBMC, without mitogen) showing less than 2 % proliferation as indicated by diminished CFSE intensity. (v) After 4 days of pPBMC stimulation with phytohemagglutinin (+PHA) typically >60 % of the CD3^+^ T cells undergo proliferation resulting in diminution of CFSE through cell division. (vi) Addition of MSPCs in a 1:3 ratio to T cells resulted in inhibition of mitogenesis-induced T-cell proliferation. Representative CD3/CFSE are shown. (C) CD3/CFSE dot plots depict representative results illustrating the gating logics for enumeration of (iv) proliferating pPBMC as a result of multiple allogeneic mixed leukocyte reactions after 7 days even without PHA and (vi) representative inhibition of proliferation in the presence of MSPC in a 1:3 ratio of MSPCs:pPBMCs ratio. (DOC 151 kb) Additional file 4: Figure S3. Thawing does not significantly hamper mean MSPC immunosuppressive function. Pooled and carboxyfluorescein (CFSE) pre-labeled random donor peripheral blood mononuclear cells (pPBMC) aliquots of 5 × 10^7^ cells were used off-the-shelf immediately after thawing in RPMI-1640/10 % pHPL and seeded in triplicate in the absence (grey boxes) or presence of phytohemagglutinin (+PHA; green boxes). Mesenchymal stem/progenitor cells (MSPCs) from each five independent bone marrow (BM), white adipose tissue (WAT) and umbilical cord (UC) donors were added immediately after thawing (dark grey area) or after a 3-day rescue culture period (light grey area). Box plots indicate median and quartiles of significant inhibition of PHA-driven T-cell proliferation by five WAT-MSPC (yellow boxes), BM-MSPC (blue boxes) and UC-MSPC (red boxes) after 4 days at an MSPC:PBMC ratio of (A) 1:3, (B) 1:10 and (C) 1:30 (except for BM-MSPC at an MSPC:PBMC ratio of 1:30). No significant difference (n.s.) was observed comparing results from freshly thawed MSPC vs. MSPC used after rescue culture for T-cell inhibition indicating overall efficiency of off-the-shelf MSPC products except for BM. Assay performance also showed absence of outliers outside the whiskers (n = 5). (DOC 84 kb) Additional file 5: Figure S4. MSPC immunosuppressive function is in part cell contact-dependent. Carboxyfluorescein (CFSE) prelabeled pooled peripheral blood mononuclear cells (pPBMC) were used after thawing in RPMI-1640/10 % pHPL in triplicate of 3 × 10^5^ cells/well in the presence of phytohemagglutinin (+PHA; A, B, C; d4) or in the absence of PHA (D, E, F; d7). Mesenchymal stem/progenitor cells (MSPC) from three randomly selected bone marrow (BM, blue bars), white adipose tissue (WAT, yellow bars) and umbilical cord (UC, red bars) donors were added 24 hours in advance to the culture vessels. (A, B, C) PHA-induced mitogenesis in the absence (green bars) or presence of MSPCs in serial dilution as indicated was measured as percentage of proliferating CD3^+^ T cells. Inhibition of T-cell proliferation by decreasing numbers of MSPCs was determined in 96-well flat-bottomed plates in direct cell–cell contact between MSPCs and pPBMCs (left part of the graphs) as compared to cell contact-independent co-cultures with MSPCs in the lower compartment and CFSE-labeled pPBMCs in the transwell insert (grey area, right part of the graphs) using (A) BM-derived, (B) WAT-derived and (C) UC-derived MSPCs as regulatory cells in serial dilution as indicated by the MSPC:PBMC ratios on the x-axis indicating significant inhibition of T-cell proliferation by all three MSPC sources at most but not all ratios but significantly reduced in transwell assays for most ratios. (D, E, F) At day 7, the potent allogeneic MLR of the pPBMCs derived from pooling ten random donor-derived cells (grey bars) in the absence of external stimulation was significantly inhibited by all three MSPC sources at some but not all ratios tested. MSPCs were tested in direct cell–cell contact between MSPCs and pPBMCs (left part of the graphs) as compared to cell contact-independent co-cultures (transwell) as described above, accordingly. Significant difference are indicated (**p* < 0.05; ***p* < 0.01; ****p* < 0.001; *****p* < 0.0001; n = 2). (DOC 259 kb)

## Abbreviations

7-AAD^−^: 7-aminoactinomycin-D-excluding
BM: Bone marrow
CFSE: Carboxyfluorescein succinimidyl ester
MHC: Major histocompatibility
MLR: Mixed leukocyte reaction
MSPC: Mesenchymal stem/progenitor cell
PBMC: Peripheral blood mononuclear cell
PHA: Phytohemagglutinin
pHPL: Pooled human platelet lysate
SD: Standard deviation
UC: Umbilical cord
WAT: White adipose tissue
