# Supplementary material for: A robust potency assay highlights significant donor variation of human mesenchymal stem/progenitor cell immune modulatory capacity and extended radio-resistance
Source: Stem Cell Res Ther. 2015 Dec 1;6:236. doi: 10.1186/s13287-015-0233-8 (PMC4666276; doi:10.1186/s13287-015-0233-8)
Supplement: Additional file 1: Table S1. — MSPC Donor age and origin. (DOC 63 kb) [file 13287_2015_233_MOESM1_ESM.doc]

**Table S1: MSPC Donor age and origin**


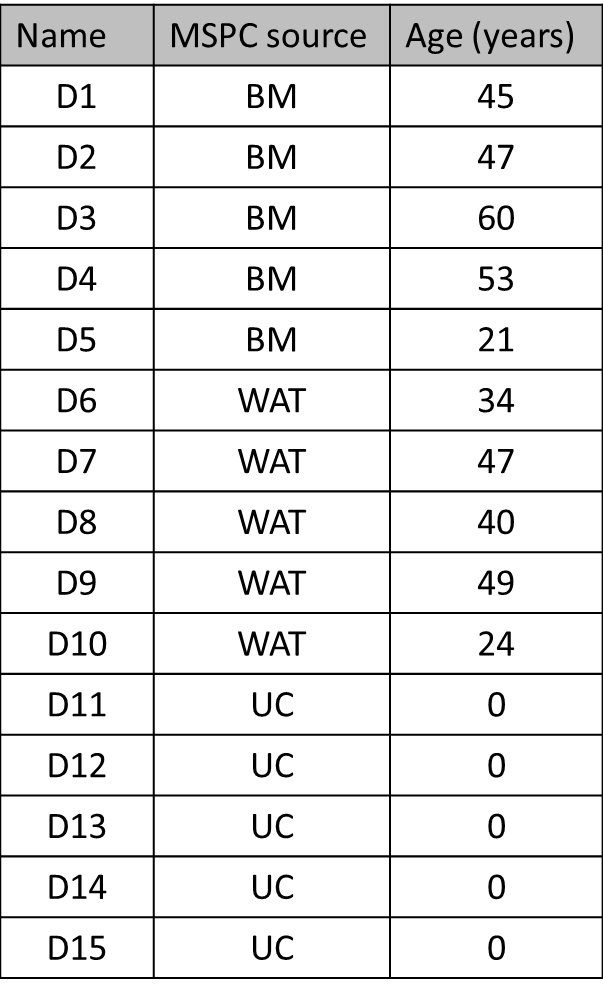


Abbreviations: bone marrow (BM), white adipose tissue (WAT), umbilical cord (UC)
