# Supplementary material for: A robust potency assay highlights significant donor variation of human mesenchymal stem/progenitor cell immune modulatory capacity and extended radio-resistance
Source: Stem Cell Res Ther. 2015 Dec 1;6:236. doi: 10.1186/s13287-015-0233-8 (PMC4666276; doi:10.1186/s13287-015-0233-8)

Ketterl et al.   Figure S3

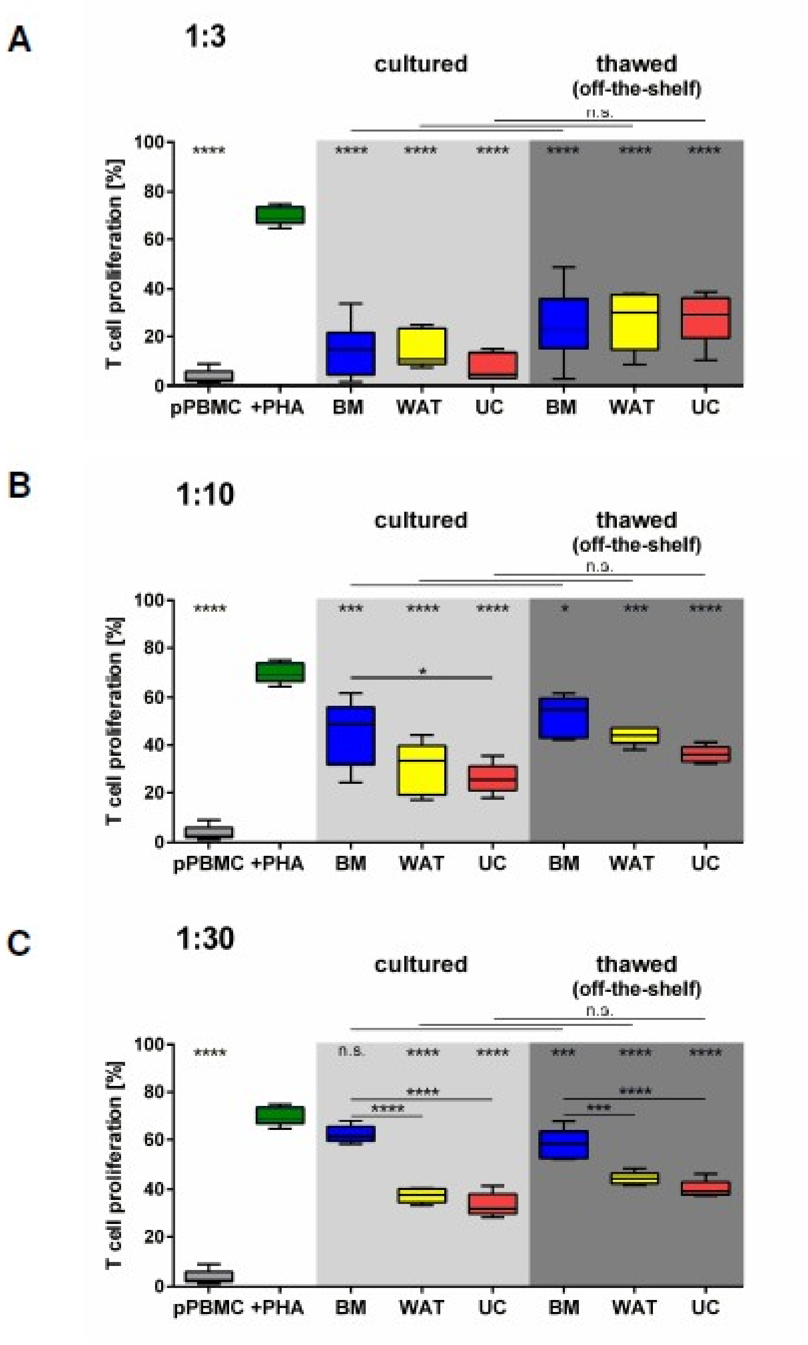

Supplement: Supplementary file 4 [file 13287_2015_233_MOESM4_ESM.doc]
